# Environmental implications of Ptolemaic Period rodents and shrews from the Sacred Falcon Necropolis at Quesna, Egypt (Mammalia: Muridae and Soricidae)

**DOI:** 10.1186/s12862-022-02101-x

**Published:** 2022-12-23

**Authors:** Neal Woodman, Salima Ikram, Joanne Rowland

**Affiliations:** 1 U.S. Geological Survey, Eastern Ecological Science Center at Patuxent Research Refuge, Laurel, MD USA; 2 Department of Vertebrate Zoology, National Museum of Natural History, Smithsonian Institution, Washington, DC USA; 3 Department of Sociology, Egyptology, and Anthropology, American University in Cairo, New Cairo, Egypt; 4 Department of Ancient Studies, Stellenbosch University, Stellenbosch, South Africa; 5 Department of Archaeology, School of History, Classics, and Archaeology, The University of Edinburgh, Edinburgh, Scotland

**Keywords:** *Acomys cahirinus*, Animal mummy, *Arvicanthis niloticus*, Climate change, *Crocidura floweri*, *Crocidura olivieri*, *Crocidura religiosa*, *Gerbillus*, Horus, *Mus musculus*

## Abstract

**Background:**

Assemblages of mummified and preserved animals in necropoleis of Ptolemaic Period Egypt (ca. 332–30 BC) document some aspects of the ceremonial and religious practices of the ancient Egyptians, but study of these animal remains can also provide insight into the local environments in which the animals and humans lived.

**Results:**

Excavations of the Sacred Falcon Necropolis at Quesna in the Nile Delta have yielded many thousands of animal remains, mostly of raptors, but also of a lesser number of small, wild mammals. Among the latter, we identified four species of murid rodents (Rodentia: Muridae) and five species of shrews (Eulipotyphla: Soricidae). The soricids are of particular interest because they represent a more diverse assemblage of species than occurs in the delta today. They include one species, *Crocidura gueldenstaedtii* (Pallas, 1811), that no longer occurs in the delta and another, *C. fulvastra* (Sundevall, 1843), that is now extirpated from Egypt.

**Conclusions:**

The coexistence of this diverse small mammal community suggests that a greater availability and variety of mesic habitats were present during the Ptolemaic Period than occur there now. The local mammal faunas recovered at Quesna and other well-studied ancient Egyptian sites together provide evidence of a richer, more complex regional environment along the Nile Valley. They also provide important insight regarding the biogeography of the individual species comprising the faunas and about the extent of faunal turnover since the Ptolemaic Period.

**Supplementary Information:**

The online version contains supplementary material available at 10.1186/s12862-022-02101-x.

## Background

Ceremonial mummification and burial of animals in ancient Egypt dates to at least 3100 BC and eventually led to the development of dedicated animal tombs during the Late (ca. 712–332 BC), Ptolemaic (ca. 332–30 BC), and Early Roman (30 BC–AD 250) periods [1–5]. Such necropoleis were typically associated with religious cults that focused on animals associated with one or more specific deities. Animals that were commonly attributed such religious significance included canids, felids, ibises, and raptors, whose cumulative remains in a single necropolis might number in the hundreds of thousands [6–10]. Among the less common remains recovered from these tombs are those of small mammals, most notably shrews (Eulipotyphla: Soricidae), but occasionally rats and mice (Rodentia: Muridae) as well. By the time of the New Kingdom (1550–1069 BC), shrews were associated with the falcon-headed god Horus, representing the god’s dark (nighttime) aspect, in contrast to the light (daytime) aspect represented by diurnal raptors [2, 4, 10–14].

Excavations of the Sacred Falcon Necropolis at Quesna in the Nile Delta have yielded many thousands of animal remains, mostly of raptors. A small percentage of the remains, however, are from small mammals that include five species of shrews and four species of murid rodents. The shrews are of particular interest because, in addition to their religious significance, they are evidence of the existence of a more abundant and diverse small mammal community in the Nile Delta during the Ptolemaic Period than is extant today. The local abundance and species richness of shrews is typically higher in mesic habitats than in xeric habitats [15], yet the fauna recovered from Quesna was preserved in the midst of a long-term period of regional climatic change leading to desertification that began ca. 5500–5000 years ago [16–18]. The ancient association of species at Quesna provides information about local conditions in the Nile Delta during the Ptolemaic Period, which may, in turn, provide insight regarding the nature of subsequent environmental changes.

In this paper, we identify the soricids and murids whose remains were excavated from the Sacred Falcon Necropolis at Quesna. We compare the small mammal community preserved there with the modern fauna and with faunas preserved at other ancient Egyptian sites, and we discuss the environmental implications of the species present.

## Methods

### Locality

**Fig. 1:**
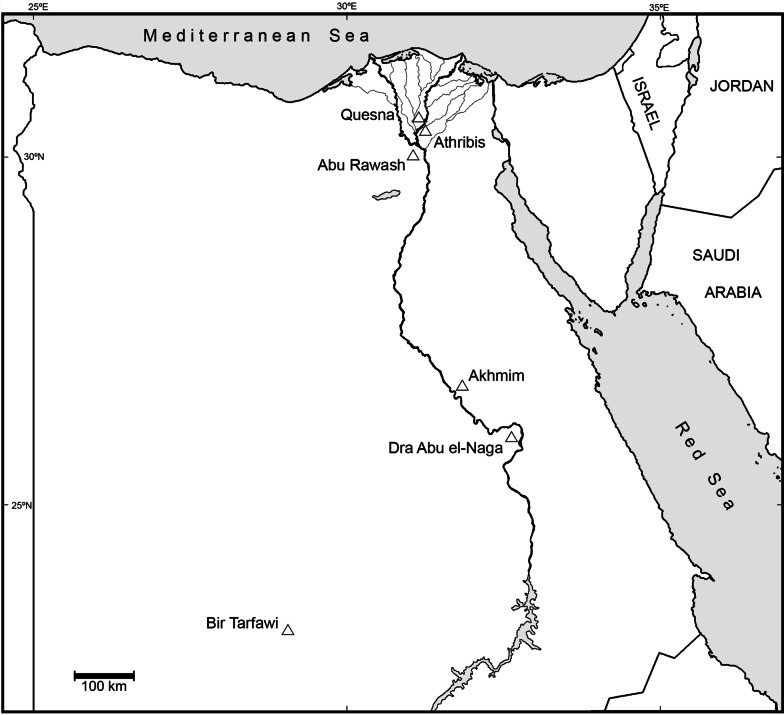
Map of Egypt showing the location of Quesna and other sites mentioned in the text

The Quesna archaeological site (30° 31′ 54″ N, 31° 10′ 18″ E) is situated on a low, sandy hill, or “turtleback,” ca. 3.5 km east of the modern town of the same name in Minufiyeh Governorate, Egypt (Fig. 1). A particular focus of the Quesna excavations has been a collapsed and buried mud-brick hypogeum on the southern edge of the turtleback identified as the Sacred Falcon Necropolis. Used as a ceremonial repository for animal mummies dedicated to the god Horus [10, 19], this structure lies close to the northern edge of an extensive Ptolemaic and Roman cemetery and directly south of a 3rd Dynasty mastaba tomb. Inscriptions from the hypogeum indicate links between Quesna and Arthribis (modern Tell Atrib, Benha), seven kilometers to the south, as early as the Late Period; texts on mud seal impressions from recent excavations in the Falcon Necropolis connect Quesna with Athribis during the Ptolemaic Period as well [10]. Athribis is the site from which Djedhor the Savior, the priest of the cult of the raptor god Horus Khenty-Khety, is known [10, 20–22]. Texts thought to originate in Athribis [20, 21] suggest the hypogeum was constructed during the time of Philip Arrhideaus (332–323 BC) at the beginning of the Ptolemaic Period and subsequently expanded.

The Sacred Falcon Necropolis was first located and partly excavated during investigations of the Quesna archaeological site by Egypt’s Supreme Council for Antiquities (SCA) in the 1990s. Following the results of geophysical surveys using magnetometry (in 2006) and ground-penetrating radar (in 2009) [23], five exploratory trenches (T2, T3, T12, T13, T15) were opened on the site during 2007–2014 [10]. Trenches T2 and T3 investigated the southern and northern parts, respectively, of the westernmost extent of the structure, which is considered to have been the main entrance to the galleries of the hypogeum that was accessible from the south. Trenches T12 and T13 explored the southernmost mud-brick corridors whose presence was suggested by the geophysical survey [23]. The fifth trench (T15) was located to investigate the central area of the structure running up to the northernmost wall. An additional area, previously excavated in the 1990s by the SCA, was re-excavated in 2012 to determine the extent of the previous investigations. This last trench was divided into three contexts, designated from west to east, (SFG1), (SFG2), and (SFG3) (SFG for Sacred Falcon Gallery) [10]. Exploratory excavations of the collapsed structure revealed corridors containing ceramic vessels, fragments of bird eggs, mummified animal remains, copper alloy figures in the form of Osiris, fragments of figures of Horus (including copper alloy beaks and talons and tail feathers), and a complete figure of a shrew [10].

In addition to more-or-less intact animal mummies, large quantities of disturbed and disarticulated skeletal remains of animals, most from decomposed or otherwise damaged mummies, were recovered by screening of excavated sediments. To date, 12,279 individual remains have been identified to at least taxonomic class, and 7970 to taxonomic order [10, 24]. The majority of animal remains (> 95% of remains identified to order) are from avians, principally Falconiformes (92%) [10, 25]. The remainder are mostly from small mammals, and it is on these disarticulated remains that our current study is based. Mammals were recovered from four contexts [10]:

- Contexts (4) and (5) were distinct locations along the south wall of T2, associated with the western entrance structure of the Falcon Necropolis.
- Context (213) in T15 was a silty sand deposit filling the southernmost of three parallel, corridors oriented east-to-west that represent one of the distinct building phases of the Falcon Necropolis.
- Context (SFG1) represented the western section of an area of the Sacred Falcon Gallery that was first excavated in the 1990s. Dense deposits of animal mummies (mostly avians) were discovered along the western wall of the gallery, and excavation yielded abundant disturbed remains of birds. Also recovered in (SFG1) was a copper alloy statuette of a shrew with a ventral peg, by which it was probably once attached to the top of a box containing the mummified remains of a shrew (similar to Egyptian Museum, Cairo JE 662, JE 7201).

### Identification

Remains of small mammals from the Falcon Necropolis were photographed, identified, and measured. All variables were recorded to the nearest 0.1 mm. For *Gerbillus*, we measured the greatest length and greatest posterior width of the lower first molar (M_1_), which was the most common element present. For *Acomys*, we used the greatest length and greatest width of the second upper molar (M^2^), which was the only tooth recovered from this taxon. Measurements from shrew dentaries are the length of the toothrow from the anterior extent of the fourth lower premolar (P_4_) to posterior edge of the third lower molar (M_3_) and the height of coronoid process of the dentary (Additional file 1). Images and measurements from archaeological remains were compared to modern specimens housed in the following collections (Additional file 2): Field Museum of Natural History, Chicago, IL, USA (FMNH); Natural History Museum, London, UK (NHMUK); University of Michigan Museum of Zoology, Ann Arbor, MI, USA (UMMZ); National Museum of Natural History, Washington, DC, USA (USNM); and Yale Peabody Museum of Natural History, New Haven, CT, USA (YPM).

The number of identified remains (NIR) is the total number of remains that could be identified to a particular taxon. The minimum number of individuals (MNI) for each taxon was determined by counting the most abundant element of the skull, which was typically either the left or right dentary.

## Results

### Distribution of remains

We recovered 367 identifiable mammalian skeletal elements from four archaeological contexts at Quesna (Table 1). More than half of the remains (56%) are from soricids, the remainder are from murid rodents. The largest number of mammalian remains (*n* = 184) was recovered from Context (213), which contained only shrews.

**Table 1:** Numbers of identified remains (NIR) of mammals from four archaeological contexts associated with the Sacred Falcon Necropolis at Quesna

	Context				Site total (*n*)	Site total (%)
	(4)	(5)	(SFG1)	(213)		
Shrews						
*Crocidura floweri/whitakeri*	2	1	–	2	5	1%
	< 2%	< 2%		1%		
*Crocidura fulvastra*	–	–	–	21	21	6%
				11%		
*Crocidura olivieri*	5	1	3	118	127	35%
	4%	< 2%	30%	64%		
*Crocidura religiosa*	2	2	7	1	12	3%
	< 2%	4%	70%	< 1%		
*Crocidura gueldenstaedtii*	–	–	–	42	42	11%
				23%		
Total shrews	9	4	10	184	207	
Rodents						
*Arvicanthis niloticus*	23	4	–	–	27	7%
	19%	7%				
*Mus musculus*	78	43	–	–	121	33%
	66%	80%				
*Acomys cahirinus*	1	–	–	–	1	< 1%
	< 1%					
*Gerbillus*	8	3	–	–	11	3%
	7%	6%				
Total rodents	110	50	0	0	160	
Total mammals	119	54	10	184	367	

Context (4), near the main entrance to the Falcon Gallery, yielded the second largest number of remains (*n* = 119) and the greatest diversity of mammals (7 species). Context (5), also near the main entrance, provided the third largest number of remains (*n* = 54) and the second greatest diversity (6 species). Mammal remains in both Contexts (4) and (5) were dominated by rodents, particularly *Mus musculus* Linnaeus, 1758.

The lowest number of mammalian remains (*n* = 10) was recovered from the previously excavated (SFG1), where fewer would be expected as a result of the earlier work there. All remains from this context were from shrews.

### Identified taxa

**Fig. 2:**
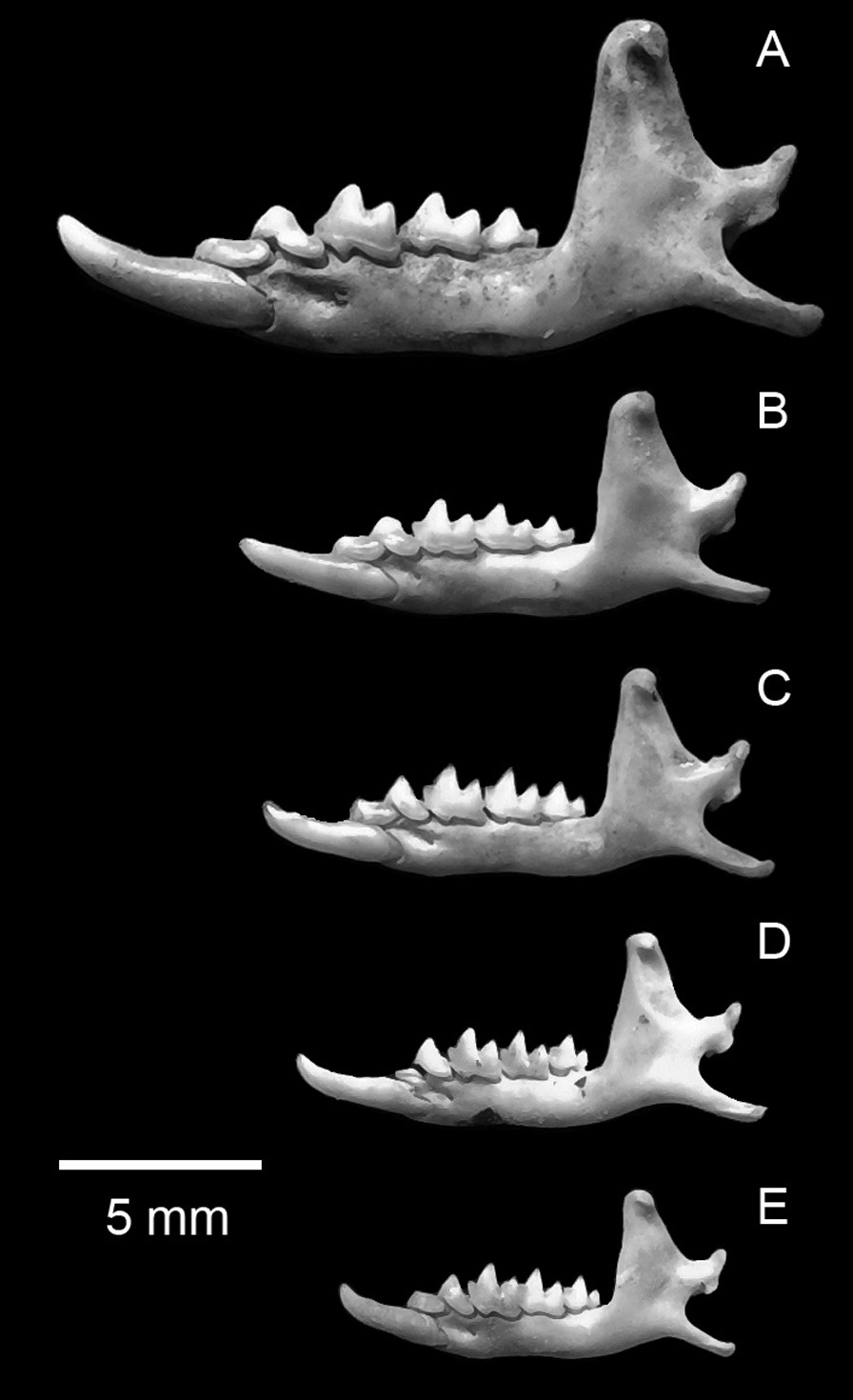
Buccal view of left dentaries of shrews from Quesna. **A** *Crocidura olivieri*; **B** *C. fulvastra*; **C** *C. gueldenstaedtii*; **D** *C. floweri* or *C. whitakeri*; **E** *C. religiosa*

**Fig. 3:**
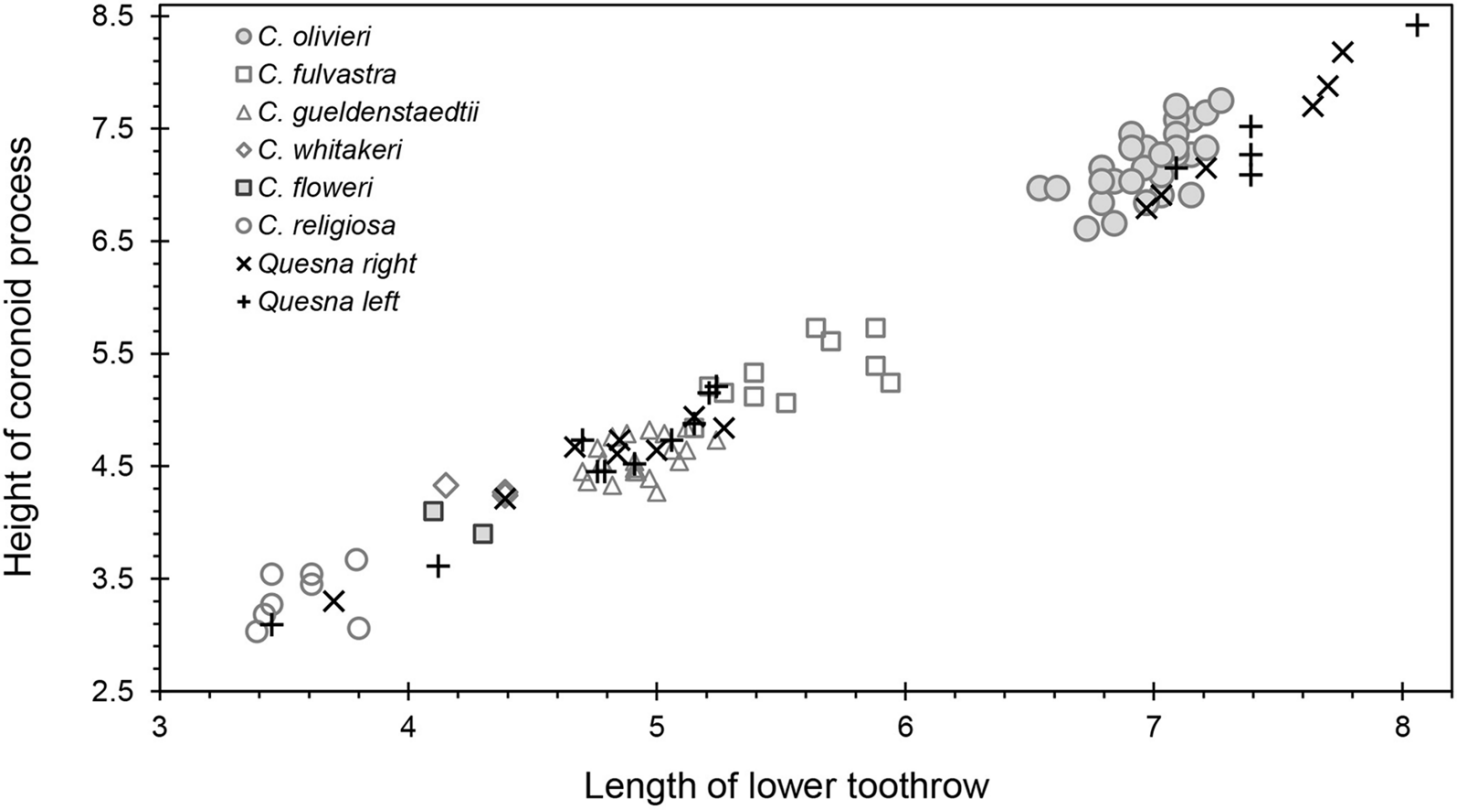
Plot of length of lower toothrow (P_4_ to M_3_) and height of coronoid process of shrew dentaries from Quesna compared with those from six species of modern shrews

**Fig. 4:**
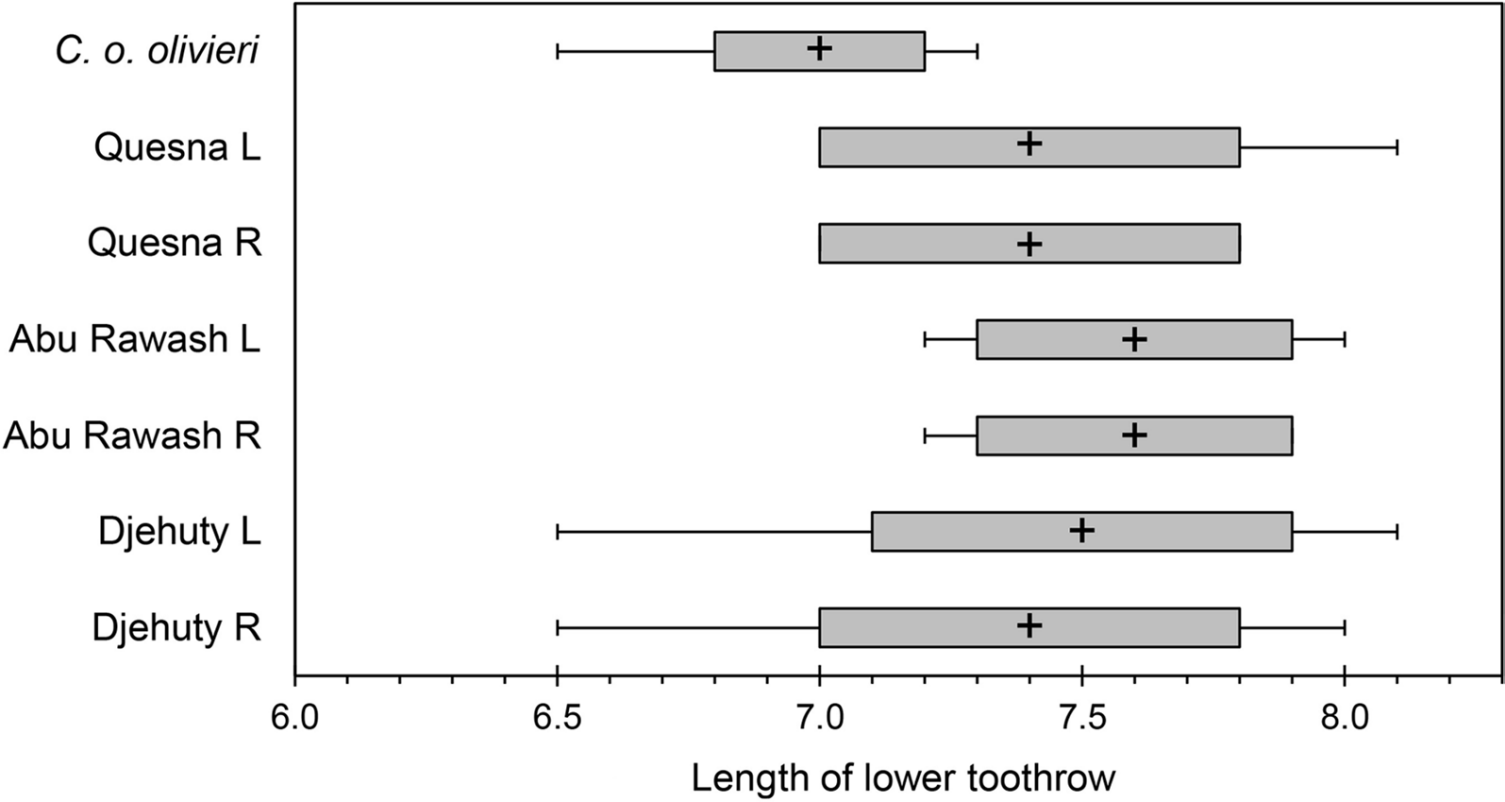
Box and whisker plot comparing lengths of lower toothrow (P_4_ to M_3_) from modern dentaries of *Crocidura olivieri olivieri* (*n* = 32) with those from ancient dentaries of *C. olivieri* from Quesna (8 left, 7 right), Abu Rawash (6 left, 4 right), and Dra Abu el-Naga (Djehuty: 19 left, 24 right). Crosses represent mean values; solid bars are two standard deviations of the mean; lines indicate the ranges of measured values. See Additional file 1: Table S2. *L* left dentary, *R* right dentary

Remains of small mammals from the Falcon Necropolis represent a minimum of 173 individuals of five species of shrews and four species of murid rodents (Table 2). The most abundant small mammal identified from the tombs was the African Giant Shrew, *Crocidura olivieri* (Lesson, 1827). Remains of this species were also among the most widespread in the Falcon Necropolis, occurring in all four archaeological contexts. This large shrew (Figs. 2A, 3) is one of the most common and abundant small mammals recovered from ancient Egyptian animal tombs (Table 3) [2, 24, 26–30]. Like *C. olivieri* from other ancient Egyptian sites, those from Quesna average larger in size than modern Egyptian populations (Fig. 4) [30, 31]. The African Giant Shrew is a widespread species complex distributed across Africa south of the semidesert zone to northern Namibia and central Mozambique. It is found in a range of habitats from evergreen forests to grasslands, and it occurs in cultivated fields and near human habitations. The species is typically common where it occurs. Genetically typical *C. olivieri* is restricted to an eastern subset of these populations, including a disjunct population in the northernmost Nile Valley and the Fayum in Egypt that is sometimes distinguished as the subspecies *C. olivieri olivieri*. The species is currently absent from the southern Nile Valley in Egypt [32–34]. *Crocidura olivieri* was referred to previously as *C. flavescens* (I. Geoffroy St.-Hilaire, 1827) [32; but see 28].

**Table 2:** Minimum numbers of individuals (MNI) of mammals calculated for four archaeological contexts associated with the Sacred Falcon Necropolis at Quesna

	Context				Site total (*n*)	Site total (%)
	(4)	(5)	(SFG1)	(213)		
Shrews						
*Crocidura floweri/whitakeri*	1	1	–	1	3	2%
	< 2%	4%		1%		
*Crocidura fulvastra*	–	–	–	9	9	5%
				11%		
*Crocidura olivieri*	3	1	2	56	62	36%
	5%	4%	33%	69%		
*Crocidura religiosa*	1	1	4	1	7	4%
	< 2%	4%	67%	1%		
*Crocidura gueldenstaedtii*	–	–	–	14	14	8%
				17%		
Total shrews	5	3	6	81	95	
Rodents						
*Arvicanthis niloticus*	9	2	–	–	11	6%
	15%	8%	–			
*Mus musculus*	42	18	–	–	60	35%
	70%	69%	–			
*Acomys cahirinus*	1	–	–	–	1	< 1%
	< 2%		–			
*Gerbillus*	3	3	–	–	6	3%
	5%	12%	–			
Total rodents	55	23	0	0	78	
Total mammals	60	26	6	81	173	
Number of species	7	6	2	5	9

**Table 3:** Assemblages of soricid and murid species from well-studied ancient Egyptian archaeological sites and their relative representation in modern Egypt

	Dra Abu el-Naga^a^	Thebes: Queen Mentuhotep^b^	Akhmim^c^	Tuna-el-Gebel^d^	Quesna	Modern Egypt^e^
Eulipotyphla: Soricidae						
*Crocidura balsamifera*^f^	–	–	3	–	–	–
*C. floweri*^g^	–	–	3	–	3	5
*C. fulvastra*^h^	1	–	2	–	9	–
*C. gueldenstaedtii*^i^	–	–	–	–	42	1
*C. olivieri*	122	2	26	38	62	104
*C. pasha*^h^	1	2	–	–	–	–
*C. religiosa*	37	15	9	15	7	18
*Crocidura* indet.	–	–	–	25	–	–
*Suncus etruscus*	–	–	7	–	–	1^j^
Rodentia: Muridae						
*Acomys cahirinus*	5	–	X^k^	–	1	275
*Arvicanthis niloticus*	9	–	–	–	11	130
*Mus musculus*	–	–	–	–	60	392
*Gerbillus* sp.	–	–	–	–	6	X
Total individuals	175	19	50	78	173	
Number of species	6	3	6	3	9	

Numerals represent numbers of individuals (MNI); “X” indicates a taxon reported as present but not quantified

^a^Woodman and Ikram [30]

^b^Woodman et al. [29]; rodents were not part of this study

^c^Hutterer [28]; rodents were not part of this study

^d^Kessler [27]

^e^Numbers are numbers of specimens inspected by Osborn and Helmy [32]

^f^Extinct

^g^Specimens from Quesna identified here as *C. floweri* may be *C. whitakeri*

^h^Extirpated from Egypt

^i^Occurs in Egypt only in the Sinai Peninsula

^j^Based on a specimen reported by Heim de Balsac and Lamotte [84]

^k^Kessler [2] reported this species as present at this site

Gueldenstaedt’s Shrew, *Crocidura gueldenstaedtii* (Pallas, 1811), was the second most abundant soricid and the third most abundant small mammal represented by remains at Quesna (Figs. 2C, 3; Tables 1, 2). This is the first and, so far, the only ancient Egyptian archaeological site from which this species has been reported, in part because of the complex taxonomic history of this and other species in the *C. suaveolens* group. Modern *C. gueldenstaedtii* has a broad distribution from western Iran, Azerbaijan, Georgia, and eastern Turkey, north of the Mediterranean Sea into western Europe and southwest along the eastern shore of the Mediterranean to Israel and the Sinai Peninsula. It is not currently known from the Nile Delta. The species is common in shrublands with well-developed stands of grass. In more arid regions, it is restricted to irrigated fields and areas near water [24, 35–38]**.** *Crocidura gueldenstaedtii* was referred to previously as *C. suaveolens portali* (Thomas, 1920) [32].

The Savanna Shrew, *Crocidura fulvastra* (Sundevall, 1843) was the third most abundant soricid recovered at Quesna, but all remains of this species were found exclusively in context (SFG1) in the westernmost portion of the Falcon Gallery (Figs. 2B, 3). The Savanna Shrew has been identified previously from ancient Egyptian animal tombs at Akhmim and Dra Abu el-Naga [28, 30]. *Crocidura fulvastra* currently has a discontinuous distribution in dry savanna habitats across central Africa, from Mali east to Ethiopia and northern Kenya, but it does not occur in Egypt. The closest modern locality for *C. fulvastra* is in southern Sudan [36, 39, 40]. Its discovery at Quesna and other archaeological sites indicates that its distribution extended up through the Nile Valley to the delta during the Ptolemaic Period [30].

The Sacred Shrew, *Crocidura religiosa* (É. Geoffroy Saint-Hilaire, 1827), is one of the more common small mammals reported from ancient Egyptian archaeological sites (Figs. 2E, 3). It has been identified in numerous ancient animal tombs in the Nile Valley [2, 26–30] and in the Old Kingdom (2575–2125 BC) settlement of ‘Ain el-Gazzareen in the Dakhleh Oasis of the Western Desert [41]. The Sacred Shrew is typically one of the more abundant small mammal species in Egyptian archaeological sites (Table 3). At Quesna, *C. religiosa* was one of only two species whose remains were recovered from all four archaeological contexts, although it was only the sixth most abundant of nine species of small mammals. The Sacred Shrew is an Egyptian endemic. Individuals have been found in cultivated fields and along canal banks in the northern Nile Valley from Luxor north to the delta [32, 36, 42, 43]. It is a very small shrew, and its ecology and habits are poorly known. *Crocidura religiosa* was incorrectly referred to previously as *C. nana* Dobson, 1890 [2, 32].

The least abundant shrew at Quesna is a tiny species that is either Flower’s shrew, *Crocidura floweri* Dollman, 1915, or Whitaker’s Shrew, *C*. *whitakeri* de Winton, 1887 (Figs. 2D, 3). Only three individuals of this shrew were identified, each from a different context (Tables 1, 2). Flower’s shrew and Whitaker’s Shrew differ in some cranial proportions, but there are too few modern specimens to adequately gauge the range of variation of either species, and the material from Quesna did not preserve characters adequate to identify the taxon with certainty. Modern *C. floweri* is an uncommon and poorly documented Egyptian endemic whose known distribution is restricted to the Nile Delta, Wadi el Natrun, and the Fayum, where it has generally been found in agricultural fields [32, 36, 44]. Its geographic distribution extended farther south in the past, as evidenced by remains of the species reported from ancient animal tombs at Akhmim [28, 31]. *Crocidura whitakeri* is distributed in coastal regions of Western Sahara, Morocco, Algeria, and Tunisia, with disjunct populations along the Mediterranean coast of Egypt west of the Nile Delta near Marsa Matruh and in the northern Sinai along Lake Bardawil. Its modern distribution suggests that it probably occurred more broadly in the historical past. Egyptian *C*. *whitakeri* were formerly identified as *C*. *suaveolens matruhensis* Setzer, 1960 [28, 32]. This species has not been identified from any archaeological sites in Egypt.

**Fig. 5:**
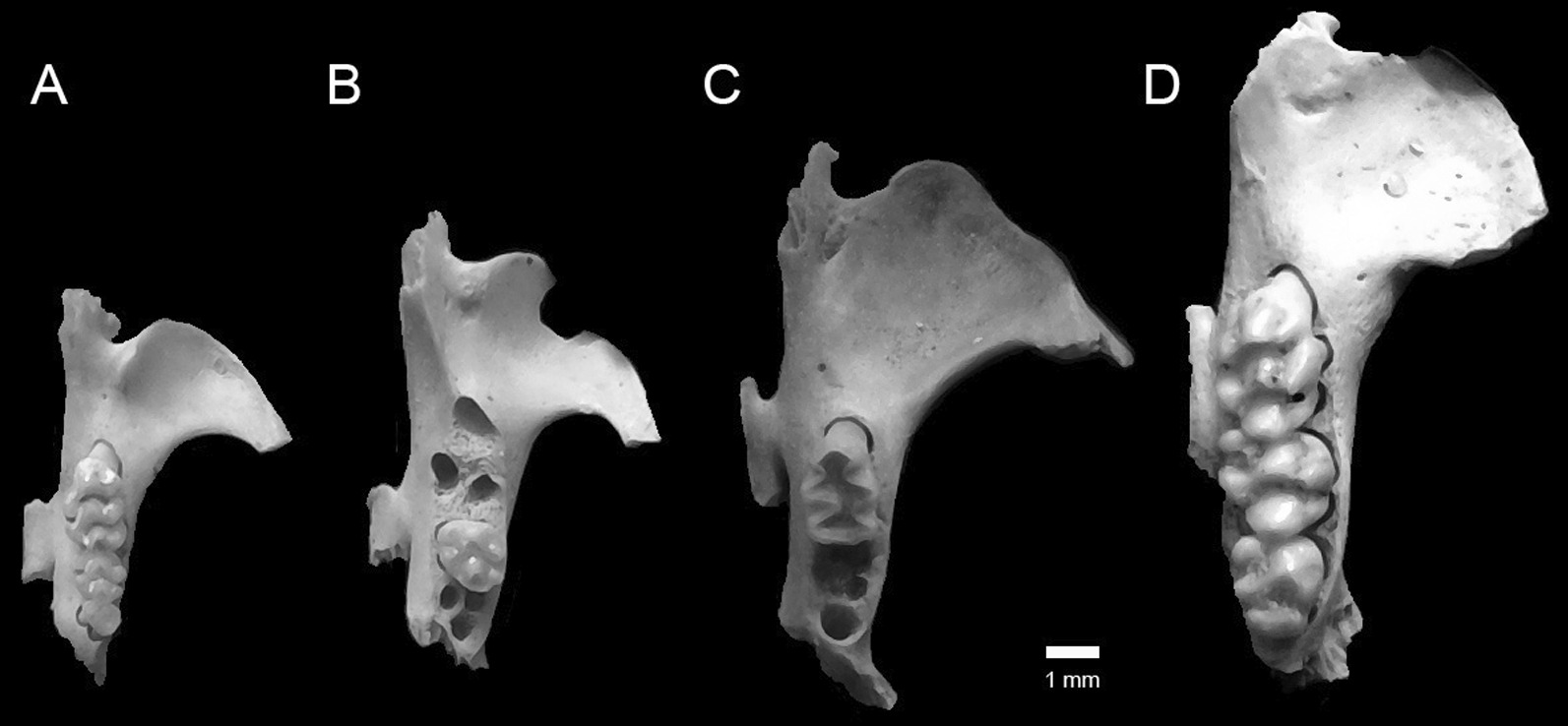
Occlusal view of left maxillary toothrows of rodents from Quesna. **A** *Mus musculus*; **B** *Acomys cahirinus*; **C** *Gerbillus*; **D** *Arvicanthis niloticus*. The maxillae are aligned by the posterior margin of the posterior alveolus of roots of the upper third molar (M^3^)

The House Mouse, *Mus musculus*, was the most abundant rodent and the second most abundant small mammal recovered at Quesna (Fig. 5A). Remains of this commensal species, and all other rodents, were limited to contexts (4) and (5) near the entrance in the far west of the Sacred Falcon Necropolis. Modern House Mice can be found in houses and nomad tents throughout Egypt, but mostly occur in the Nile Valley and Delta, in oases, and along the Mediterranean coast [32].

The Nile Grass Rat, *Arvicanthis niloticus* (É. Geoffroy Saint-Hilaire, 1803) was the second most common rodent and the fourth most common small mammal recovered from Quesna (Fig. 5D). Like the House Mouse, its remains occurred only in contexts near the western entrance to the Falcon Necropolis (Tables 1, 2). This species has been found in prehistoric and later sites [45, 46] and occasionally identified from ancient Egyptian animal burial sites [24, 27, 45, 47]. The modern Nile Grass Rat is distributed throughout the Nile Valley, from the delta to its sources and in a broad band across central Africa. It typically inhabits savannas and shrublands, and it can be a dominant species in grasslands that have sufficient cover. The Nile Grass Rat also occurs in cultivated fields, where it can be a crop pest, and in villages, although it generally does not enter human habitations [32, 48]. The species requires moisture, and local population sizes can fluctuate greatly between rainy and dry seasons and from year to year. In the Senegalese Sahel, population numbers reached ca. 100 individuals/ha following greater than average rainfall but fell to 17–18 individuals/ha in the subsequent dry season [48, 49]. The Nile Grass Rat was reportedly eaten by classical Romans [31], and there are contemporary accounts of “field-mice” or “rats” corresponding to the Nile Grass Rat being caught and eaten by some Egyptians in the late eighteenth and nineteenth centuries [50, 51]. Although Nile Grass Rats or other rodents may have been used in medicine by the ancient Egyptians, there is no clear evidence that they were part of their diet [47].

**Fig. 6:**
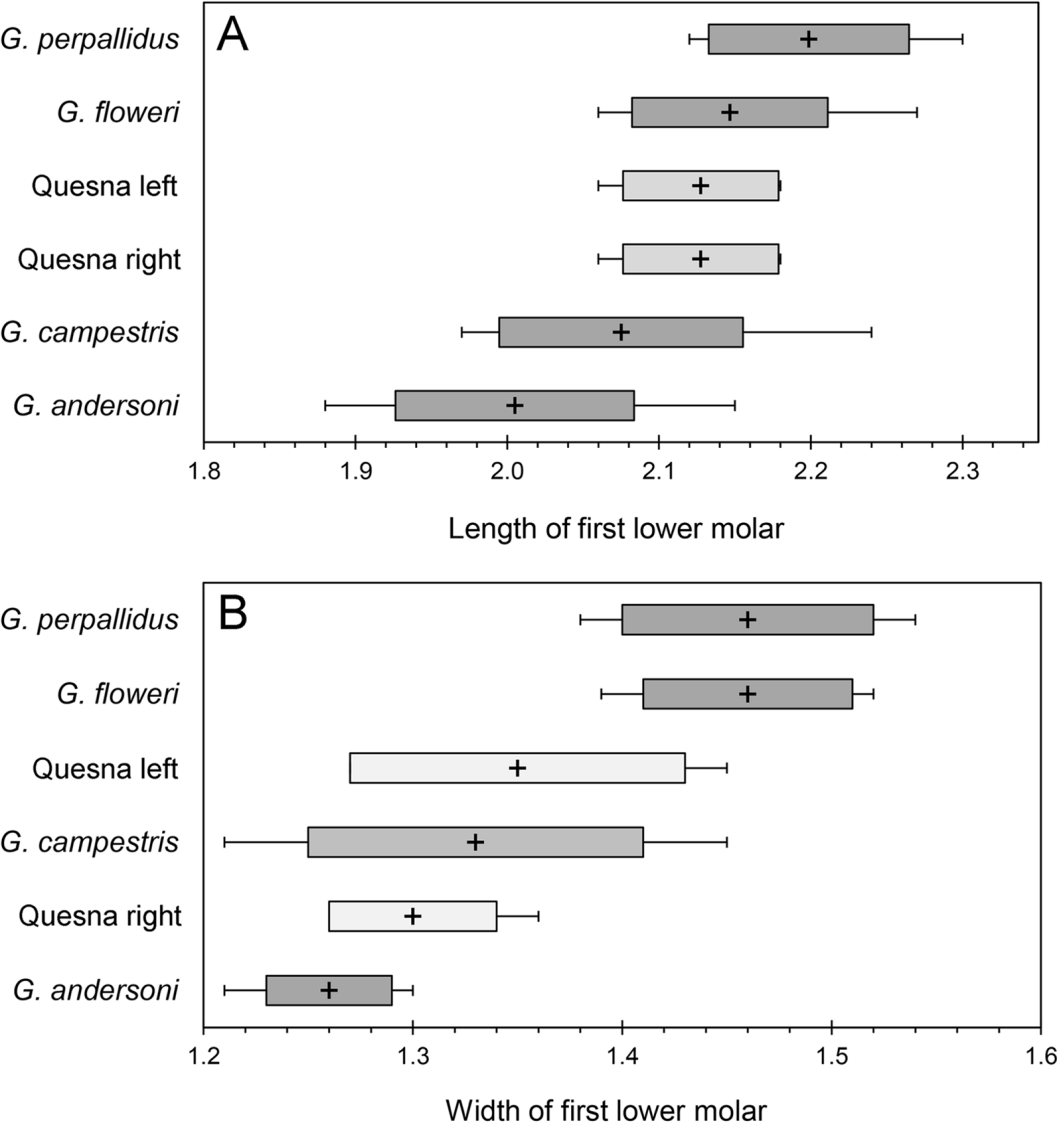
Bar and whisker plots comparing the **A** lengths and **B** widths of lower first molars (M_1_s) of *Gerbillus* from Quesna (*n* = 4) with those of modern species. Of 11 species currently known from Egypt, the Quesna molars overlap in size with only *G. andersoni* (*n* = 10), *G. campestris* (*n* = 10), *G. floweri* (*n* = 9), and *G. perpallidus* (*n* = 8), and they are closest in mean dimensions to those of *G. floweri* and *G. campestris*. Crosses represent mean values; solid bars are two standard deviations of the mean; lines indicate the ranges of measured values

The third most abundant rodent at Quesna was an undetermined species of gerbil, *Gerbillus* Desmarest, 1804 (Fig. 5C). We follow [52, 53] in recognizing *Dipodillus* Lataste, 1881 and *Hendecapleura* Lataste, 1882 as subgenera of *Gerbillus* rather than as separate genera. The range of proportions of the first lower molar (M_1_) of specimens from Quesna overlap in size with *G.* (*Gerbillus*) *andersoni* De Winton, 1902, *G.* (*Dipodillus*) *campestris* (Levaillant, 1857), *G.* (*Gerbillus*) *floweri* Thomas, 1919 (previously considered a subspecies of *G. pyramidum* I. Geoffroy Saint Hilaire, 1825 [32]), and *G.* (*Gerbillus*) *perpallidus* Setzer, 1958 (Fig. 6). All four species have mostly northern Egyptian distributions in regions with > 25 mm of annual rainfall. They can inhabit sandy dunes, but also occur in or adjacent to cultivated fields. Some occur on rocky slopes, coastal desert, muddy lake shores, or palm groves [32]. Only *G. andersoni* and *G. floweri* occur in the delta today, and only *G. andersoni* is widespread there.

**Fig. 7:**
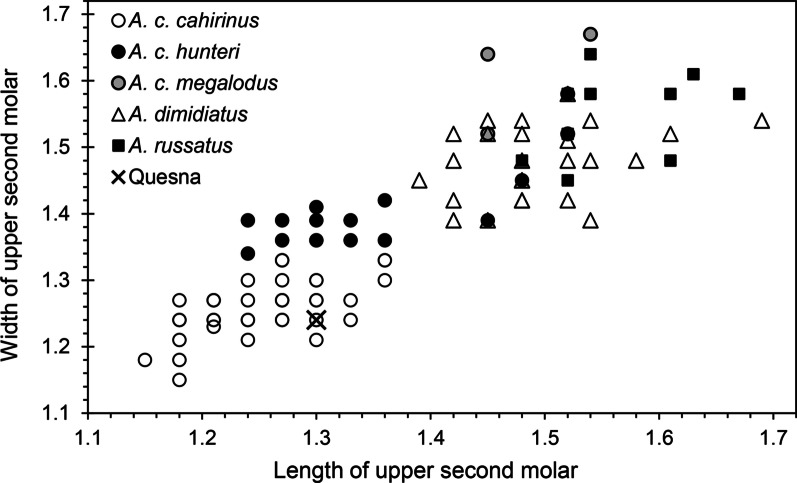
Graph of lengths and widths of the upper second molar (M^2^) of *Acomys* from Quesna (*n* = 1) with those of modern taxa. See text for explanations of taxa

A single partial left maxilla with an intact upper second molar of the Cairo Spiny Mouse, *Acomys cahirinus* (É. Geoffroy Saint-Hilaire, 1803), was recovered from context (4), near the entrance to the Falcon Necropolis (Fig. 5B; Tables 1, 2). This species has been reported from several ancient Egyptian sites with animal mummies, but its remains are not typically found in abundance [2, 24, 47]. The taxonomy of modern *Acomys* I. Geoffroy Saint-Hilaire, 1838 is complex, with 21 recognized species [54] and at least 26 genetic clades warranting integrative taxonomic analyses [55]. Three species or clades are known to occur in Egypt. The Eastern Spiny Mouse, *A. dimidiatus* (Cretzschmar, 1928), occurs throughout the Sinai Peninsula, with a discontinuous distribution in the Middle East as far east as southern Pakistan [56–58]. The Golden Spiny Mouse, *A. russatus* (Wagner, 1840), occurs in Egypt east of the Nile and in the Sinai Peninsula, as well as on the Arabian Peninsula and east as far as Jordan and Lebanon [56, 59]. The Northeast African Spiny Mouse, *Acomys cahirinus*, is common and has a widespread but discontinuous distribution across much of North Africa, including most of Egypt and parts of the eastern Sinai Peninsula [57, 61]. *Acomys cahirinus* may not be monotypic. In addition to the nominal form, five subspecies of *A. cahirinus* previously were recognized in Egypt [32]. One of these, *A. dimidiatus* is now considered a distinct species, but the other four currently are not recognized [56]. We discovered, however, that three subspecies broadly correspond to the size variation we observed in dental measurements, and we employ the names provisionally as follows: *A. c. cahirinus* for the dentally smallest modern form that inhabits the Nile Valley and delta; *A. c. hunteri* (De Winton, 1901) for a form with the lower second molar (M_2_) intermediate in size between *A. c. cahirinus* and *A. dimidiatus* and inhabiting much of the eastern desert; and *A. c. megalodus* Setzer, 1959 for the population having M_2_ larger than that of *A. dimidiatus* but averaging smaller than that of *A. russatus* and occupying the northern part of the eastern desert (Fig. 5). Amongst these taxa and potential taxa, the single specimen from Quesna compares most favorably in size with *A. c. cahirinus* (Fig. 7), which is restricted to the Nile Delta [32]. Modern *A. cahirinus* occurs on hillsides, cliffs, and other rocky habitats throughout Egypt. It is also found in human structures, including houses, outbuildings, temples, and tombs, and it will live commensally with humans. Although generally considered herbivorous and granivorous, the Cairo Spiny Mouse is also opportunistically omnivorous, eating a variety of insects, spiders, and snails [32, 60]. It was also reported consuming dried flesh and bone marrow of mummies entombed at Gebel Drunka [32].

## Discussion

### Origin of the mammal remains

During the 30th Dynasty-early Ptolemaic Period, the Falcon Necropolis at Quesna was constructed as a sacred repository for mummified remains of animals dedicated to the god Horus Khenty-khety. The majority of these remains are from raptors and represent votive mummies that probably served as messengers from individuals in the physical world to the falcon-headed god in the spiritual world [4]. Shrews were similarly associated with Horus, representing the nighttime aspect of the god, in contrast to the daytime aspect represented by diurnal raptors [2, 4, 10–14].

The much lower numbers of remains of shrews relative to those of raptors at Quesna may imply something about the relative significance of the light vs. the dark aspects of Horus to the concerns of the ancient Egyptian people, or it may be specific to the people living in Athribis and around Quesna. It may also reflect the greater availability of raptors vs. shrews in the area. While it is possible that the ancient Egyptians raised shrews in captivity for ceremonial purposes, as they did for other animals associated with deities [4, 13, 14, 25, 26, 61–64], the potential difficulties inherent in maintaining the relatively high diversity and relatively low numbers of soricids used for religious purposes would seem to make this practice economically impractical [30]. It is more likely that soricids were obtained as by-catch of active trapping for rodent pests [54, 65] and, as suggested by Rainer Hutterer, as prey of domestic cats [29]. The remains of these small mammals signify that habitats necessary to sustain viable populations of shrews existed nearby.

In contrast with shrews, rodents had no clear association with any of the ancient Egyptian gods [4, 66, 67] and were more generally viewed as pests [55, 68]. Despite the negative associations, some rats and mice were deliberately mummified and ceremonially interred, possibly considered as acceptable substitutes for similarly sized shrews when the latter were difficult to obtain and in short supply [26, 29, 69–71]. Other rodents were introduced accidentally or intruded on their own into sarcophagi or tombs [72]. Regardless, rodent remains are generally present in low abundance relative to those of soricids. At Quesna, however, recovered rodent remains were nearly as abundant as those of shrews, particularly remains of the House Mouse, which was the second most abundant species of small mammal. Yet, rodents were restricted in their distribution at the site, as they were recovered only in the vicinity of the main entrance to the Falcon Necropolis. The location of House Mouse remains suggests that, rather than being part of the mummified fauna, they may represent either a deposit below a former feeding roost for owls or raptors, or a small population of House Mice living in or near the entrance and possibly feeding on food offerings or even on mummified remains, as has been reported elsewhere for modern spiny mice [32]. In either case, the rodents, too, were part of the local fauna.

### The House Mouse in ancient Egypt

Modern House Mice in southern Europe, the Middle East, and north Africa are recognized as a genetically identifiable subspecies, *Mus musculus domesticus* Schwarz & Schwarz, 1943 [73]. This taxon is thought to have differentiated in the Iranian Plateau by the Middle Paleolithic [74–76] or ca. 478,500–236,500 yr BC [77]. *Mus m. domesticus* subsequently diffused westward into the eastern Mediterranean basin, where it developed its niche as a human commensal with the early Natufian culture in the southern Levant ca. 13,000 yr BC [78]. A second wave of westward colonization into western Mediterranean regions of southern Europe is thought to have occurred during the 1st millennium BC [79, 80].

The timing of western colonization by the House Mouse south of the Mediterranean remains speculative, as there is surprisingly little clear documentation of the House Mouse from ancient Egyptian sites [79]. It was speculated [81] that “abundant rodent tunneling” reported at Kahun (ca. 1700 BC) in the Fayum [82] and “rodent remains” noted at Buhen (ca. 1700 BC), just below the second cataract of the Nile [83], indicated the presence of the House Mouse at those sites. More direct evidence, however, indicates the House Mouse may have occupied the Nile Valley and its delta for an even longer period of time. Skeletal remains identified as *M. musculus* were reported from Neolithic (ca. 3000 yr BC) sediments at Merimde Beni-Salama in the delta, in the Old Kingdom settlement of ‘Ain el-Gazzareen in the Dakhleh Oasis of the Western Desert, 5th and 6th Dynasty (2450–2175 BC) graves at Elephantine along the upper Nile and at Saqqara, and from Middle Kingdom (2110–1630 BC) layers at Tel el Maskhuta in the delta [40, 44, 47]. These earlier records render the presence of *Mus musculus* at younger sites, such as Quesna, less surprising.

### Environmental significance of the mammal fauna

The four taxa of rodents preserved in the Ptolemaic fauna at Quesna are widespread in Egypt, although all are associated with moister regions of this mostly desert country. *Acomys cahirinus cahirinus* is restricted to the Nile Delta, *Arvicanthis niloticus* inhabits the Nile Valley and desert oases, the likely species of *Gerbillus* occur in the areas of higher rainfall of northern Egypt, and *Mus musculus* is a human commensal [32]. All occur in or near the Nile Delta today. Overall, their presence at Quesna is not particularly informative in regard to the environment, except to suggest that the moisture in the Nile Valley was no more limiting then than it is presently.

The modern fauna of Egypt includes a total of five shrew species. *Crocidura olivieri* is relatively common, but the remaining species are now uncommon to rare. An approximate idea of the relative abundances of these species in modern Egypt can be estimated from [32], in which the accounting of available museum specimens, like the fauna in the Falcon Necropolis, represents a time-averaged accumulation (Table 3). The modern survey included 104 specimens of *C. olivieri* (as *C. flavescens deltae*) from 25 localities; 18 *C. religiosa* from seven localities; five *C. floweri* from two localities; and 1 *C. gueldenstaedti* (as *C. suaveolens portali*) from the Sinai Peninsula [32]. The presence of *Suncus etruscus* in Egypt is based on a single specimen from the Nile Delta rediscovered in the Muséum National d'Histoire Naturelle, Paris prior to 1957 [32, 84, 85]. The catalog number (MO-1883–625) suggests the specimen was catalogued in 1883, and the species may now be extirpated from Egypt. Hence, only three of the five shrew species are currently known in the Nile Delta.

Local species diversity and abundance of shrews is generally higher in moister habitats than in drier ones [15]. The greater species richness and the high relative abundances of the five species of shrews from the Falcon Necropolis at Quesna (Table 3) suggest greater availability and variety of suitable mesic habitats in the Nile Delta than are present in the region today. The abundances of *C. gueldenstaedti*, which no longer occurs in the delta, and *C. fulvastra*, which no longer occurs in Egypt, suggest the existence of grasslands or grassy scrubland in parts of the delta. This interpretation is consistent with moderate levels of grass and sedge pollen in Ptolemaic-age wetland sediments from Wadi el Natrun and the Burullus Lagoon [18, 86]. Although catastrophic low floods occurred during the Ptolemaic Period [87], which has been described as a time of increased seasonal rainfall in the Fayum [86], studies of microfossils, sediment compositions, and sedimentation rates in the delta indicate the Nile generally maintained a moderate flow during most of the period [18, 88–90].

Beyond the cultural and religious concerns that are more generally the focus of archaeological investigations, the study of assemblages of mammals preserved as mummies by Ptolemaic Period Egyptians has relevance both for understanding the biogeography of the species preserved and for understanding the ancient environments in which animals and humans lived. Individual species of soricids are particularly useful because their habitat requirements often limit their distributions. Eight species of shrews are now known from ancient Egyptian archaeological sites, including one extinct species and two species now extirpated from Egypt [24, 28, 29]. Lists of shrew species identified from five well-studied ancient Egyptian sites in the Nile Valley from Luxor north to the delta indicate that known species diversity at individual sites ranged from three to six species (Table 3). Only two species, *C. olivieri* and *C. religiosa*, are common to all five sites, and they are typically among the most abundant shrew species recovered from Egyptian archaeological sites. The remaining species have limited modern distributions in Egypt, and they appear to have had similarly limited distributions in the past, making them potentially valuable for understanding past moisture gradients and habitat distributions.

## Conclusions

The Sacred Falcon Necropolis at Quesna in the Nile Delta was constructed near the beginning of the Ptolemaic Period to house mummified remains of animals dedicated to the falcon-headed god Horus Khenty-khety. Excavations of the necropolis have yielded many thousands of animal remains, mostly of raptors. In addition, we identified four species of rodents, including the commensal House Mouse, *Mus musculus*, and five species of soricids that include one species that no longer occurs in the delta and another that is extirpated from Egypt. Gueldenstaedt’s Shrew, *Crocidura gueldenstaedtii*, ranged farther west along the Mediterranean Coast during the Ptolemaic Period, and the Savanna Shrew, *C. fulvastra*, had a distribution that extended the length of the Nile Valley. The relatively diverse assemblage of shrews, which require moist conditions, suggests the presence of a variety of more mesic habitats than currently occur in the Nile Delta. Turnover in the small mammal community from the Ptolemaic Period to the present has accompanied a continuation of a long-term trend of regional desiccation previously documented by historical and geological evidence.

## Supplementary Information

**Additional file 1.** Tables of measurements used: **Table S1.** Measurements of *Crocidura* for Fig. 3. **Table S2.** Measurements of *Crocidura olivieri* for Fig. 4. **Table S3.** Measurements of *Gerbillus* for Fig. 6. **Table S4.** Measurements of *Acomys* for Fig. 7.

**Additional file 2.** List of modern specimens examined.

## Data Availability

All data generated or analyzed during this study are included in this published article and its Additional files.

## Abbreviations

BC: Before Christ
AD: Anno Domini
FMNH: Field Museum of Natural History, Chicago, IL, USA
MNI: Minimum number of individuals
NHMUK: Natural History Museum, London, UK
NIR: Number of identified remains
SCA: Supreme Council for Antiquities
SFG: Sacred Falcon Gallery
UMMZ: University of Michigan Museum of Zoology, Ann Arbor, MI, USA
T: Excavation trench
USNM: National Museum of Natural History, Washington, DC, USA
YPM: Yale Peabody Museum of Natural History, New Haven, CT, USA
