# Supplementary material for: Environmental implications of Ptolemaic Period rodents and shrews from the Sacred Falcon Necropolis at Quesna, Egypt (Mammalia: Muridae and Soricidae)
Source: BMC Ecol Evol. 2022 Dec 23;22:148. doi: 10.1186/s12862-022-02101-x (PMC9789621; doi:10.1186/s12862-022-02101-x)
Supplement: Supplementary file 2 — Additional file 2. List of modern specimens examined. [file 12862_2022_2101_MOESM2_ESM.docx]

**Additional File 2**

**List of modern specimens examined**

From: Neal Woodman, Salima Ikram, and Joanne Rowland. Environmental implications of Ptolemaic Period rodents and shrews from the Sacred Falcon Necropolis at Quesna, Egypt (Mammalia: Muridae and Soricidae).

Institutional abbreviations: FM: Field Museum of Natural History, Chicago, IL, USA; NHMUK: Natural History Museum, London, UK; UMMZ: University of Michigan Museum of Zoology, Ann Arbor, MI, USA; USNM: National Museum of Natural History, Washington, DC, USA; YPM: Yale Peabody Museum of Natural History, New Haven, CT, USA.

Eulipotyphla, Soricidae:

***Crocidura floweri*** (*n* = 7).⸻Egypt: Beheira Governate: Wadi el Natrun, 5.5 km N Lake el Zugun (UMMZ 165633, 165633). Giza Governate: Giza (NHMUK 10.6.18.2, 10.6.18.3⸻holotype; 10.6.18.6; 10.6.18.7); Kafr el Sheikh Governate: 1 km S Baltim Beach (FM 106469).

***Crocidura fulvastra*** (*n* = 28).⸻South Sudan: Al-Istiwa'Iyah Ash-Sharqiyah, Kapoeta (USNM 317888); Upper Nile Province: Paloich, Niayok (FM 93701, 93702, 93704, 93706, 93709; USNM 325928, 325929, 325930, 325931, 325932); Paloich, 1.6 km N Niayok (FM 93712); Paloich, 19 km N Niayok (FM 93711; USNM 325933); Paloich, Tir, Paloi (FM 93843; USNM 325934); Paloich, 1.6 km NE Tir (FM 93844); Paloich, 3.2 km N Tir (FM 98973); Paloich, 8 km NE Tir (FM 93846; USNM 325935); Paloich, Tir, Toich, Thaak (FM 93848; USNM 325936, 325937); Paloich, Tir, Toich, 3.2 km S Thaak (FM 93851); Paloich, Kum Kum Forest (FM 96213). Sudan: Southern Kordofan Province: Buk (FM 29008). Eastern Equatoria Province: Kapoeta (FM 85173). Kassala Province: Aroma Madart, on River Gash, 21 km SSE of Kassala (USNM 325927).

***Crocidura gueldenstaedtii*** (*n* = 24): Iran: Azarbaijan-E Gharbi Province: 11 km N Rezaiyeh, (USNM 354509); Azarbaijan-E Sharqi Province: 5 km SE Meyaneh (USNM 354503, 354504, 354505, 354514, 354517, 354519). Lorestan Province: 50 km SW Borujerd (USNM 350117). Kermanshah Province: 42 km W Kermanshah (USNM 354501, 354502, 354507, 354508, 354511). Turkey: Icel Province: Tarsus (USNM 327226, 327227, 327228, 327229); 8 km S Namrun, on Cehennem Dere (USNM 327230, 327231, 327232, 327233, 327234, 536423, 536424).

***Crocidura olivieri olivieri*** (88).—Egypt: no locality (FMNH 98221). Cairo: El-Abaseya (FMNH 140086). Damietta: Damietta (FMNH 74556; USNM, 311742). Faiyum: Kom O'Shim Forest (FMNH 98166, 98167, 98168); Kom Aushim (YPM 11500). Gharbia: Tanta, Abu Gazia (FMNH 140087, 140088). Giza: Al Badrashayn District, Sakkara (USNM 341930); Dashur (FMNH 77322); Giza: Giza (NHMUK 4.5.15.1, 4.5.15.2, 9.7.1.14, 9.7.1.15, 9.7.1.81, 10.6.18.1; FMNH 74939, 74940, 91331; USNM 311749, 311750, 311751, 311752); Imbaba, Abu Ghalib (FMNH 74937); Imbaba, Abu Rawash (FMNH 74945, 75666, 79797, 87600, 92259, 98972, 100740, 100743, 100777, 101374, 101375, 101886, 101997, 101998; USNM 311743, 311746, 311759, 311760, 341923, 341929, 341931; YPM 2803); Imbaba, between Abu Rawash & Mansuriya (FMNH 77323); Imbaba, El Baraqil (FMNH 80524; USNM 311744, 311745); Imbaba, El Mansuriya (FMNH 74568, 74569); Imbaba, Kerdasa (FMNH 90458; USNM 341924, 341925); Giza, Imbabah District, Manshiyet Radwan (USNM 311758); Imbaba, Mansuriya (FMNH 74558; USNM 311763, 311764, 341932); Imbaba, Nahya (FMNH 74570, 91218; USNM 311748); Imbaba, Saft El Laban (FMNH 89983); Imbaba, Tanash (USNM 311747); Imbaba, Wardan (FMNH 74564, 74565, 74567, 74932, 74933, 74934; USNM 311753, 311754, 311755, 311756, 311757); Mena House NHMUK 95.9.2.2, 95.9.2.3, 95.9.2.4, 95.9.2.5, 95.9.2.6); Talbia (USNM 277293). Matruh: 3 mi N Faiyum, Royal Shooting Club (FMNH 75667, 75668; USNM, 311761, 311762).

***Crocidura religiosa*** (*n* = 28).⸻Crania: Egypt: Cairo (NHMUK 1904.8.2.4, 1904.8.2.5, 1904.8.2.6, 1904.8.2.7, 1904.8.2.8); East Bank of Nile, University Farm, Assuit (NHMUK 1976.547). Giza Governate: Giza (NHMUK 1910.6.18.4, 1910.6.18.5); Imbaba, Abu Rawash (FMNH 84653, 89580, 89985, 95891, 98169, 100739, 106443; NHMUK 1916.8.16.2, 1916.8.16.3; USNM 311766, 341933; YPM 5840); Imbaba, Minshat el Bakkari (FMNH, 84753); Imbaba, Kafr Hakim (USNM 311767); Imbaba, Nahya (FMNH, 84567; USNM 311768). Luxor Governate: Luxor Necropolis of Karnak (UMMZ 165634, 165635, 165636). No locality (FMNH, 108383).

***Crocidura whitakeri*** (*n* = 7).⸻Algeria, Ain Sefra (NHMUK 13.8.6.11, 13.8.6.12). Egypt, Matruh, 4.8 km W Marsa Matruh (FMNH 91184). Morocco: Sierzet, about halfway between Morocco city and Mogador (NHMUK 98.7.4.5⸻holotype); Safi, 5 km E Essaouira (USNM 482019, 48220, 485838).

Rodentia, Muridae:

***Gerbillus andersoni*** (10).—Egypt: Al Iskandariyah: Alexandria Area, 0.5 Mi W of Dikheila Airfield (USNM 283246, 283252, 283253, 283254); Alexandria Area, 0.5 Mi E of Muntazah (USNM 283248, 283250, 283251). Al Jizah: Abu Rawash (USNM 300220). Kafr Ash Shaykh: Baltim (USNM 300225, 300226).

***Gerbillus campestris*** (10).—Egypt: Matruh: Siwa Oasis (USNM 300239, 300240, 300241, 300242, 300243, 316642, 316643, 316644, 316645, 316646).

***Gerbillus floweri*** (9).—Egypt: Al Qalyubiyah: Khanka (USNM 316844); Kafr Abu Sir (USNM 316845, 316846). As Ismailiyah: Abu Sultan (USNM 316843). Janub Sina: El Arish (USNM 316847, 316848, 316849, 316850, 316851); Quseima (USNM 316852).

***Gerbillus perpallidus*** (8).—Egypt: Al Buhayrah: Mudiriyet El Tahrir, 2 Km W of El Birigat, Kom Hamada, Beheira (USNM 316817, 316818, 316819, 316820). Matruh: Wadi Natroun (USNM 341971). Western Desert: Burg El Arab (USNM 316821); Bir Victoria (USNM 316822, 316823).

***Acomys cahirinus cahirinus*** (35).—Egypt: Ad Daqahliyah: Simbillawein, 5 Mi W (USNM 283274, 283309). Al Iskandariyah: Alexandria (USNM 282526, 283269). Al Jizah: Cairo (USNM 283270, 283271, 283272, 283273); Imbaba, Abu Rawash (USNM 300277, 316909, 342020). Imbaba, 2 Mi W of Abu Rawash (USNM 342021); no locality (USNM 300274, 300275). Al Qahirah: Abbassia (USNM 282535, 282537, 300276, 300278, 300279, 316910, 316911, 316912, 316913, 316922); Citadel (USNM 316924, 342019); Maadi (USNM 316914, 342022). As Suways: Suez (USNM 282527, 282528, 282529, 282530, 282531, 282532, 282533).

***Acomys cahirinus hunteri*** (13).—Egypt: Al Bahr Al Ahmar: Bir Abraq (USNM 316972, 316973, 316974, 316975, 316990). Sudan Administrative Area: Bir Kansisrob (USNM 316980, 316982, 316983); Wadi Kansisrob, Jebel Elba (USNM 316985, 316986, 316987, 316988, 316989).

***Acomys cahirinus megalodus*** (5).—Egypt: As Suways: Gebel Sukhna, North Gala Range (USNM 316993); Wadi Dom (USNM 350065); Gebel El Galala El Bahariya, Wadi Sayal (USNM 316994, Gebel El Galala El Bahariya, Wadi Sayal, 316995, Gebel El Galala El Bahariya, Wadi Sayal, 316996).

***Acomys dimidiadus*** (26).—Egypt: Janub Sinai: Feiran Oasis (USNM 316944, 316945); Wadi El Arbaeen, Saint Catherine's Monastery Area (USNM 316946, 316947); Wadi El Sheikh, Saint Catherine's Monastery Area (USNM 316948, 316949, 316950, 316951). Jordan: Beit-Sahur (USNM 520972); Deir-Mar-Saba (USNM 520973); Beit-Sahur, (USNM 520974); Bethlehem (USNM 520975, 520976, 520977, 520978, 520979, 520980); 34 Km N Agaba (USNM 520981); Dead Sea, Ain Faschka (USNM 520982, 520983, 520984, 520985, 520986, 520987); 4 Km NW Beit-Fajjar (USNM 520988). Saudi Arabia: 5 mi E of Taif (USNM 297601).

***Acomys russatus*** (12).—Egypt: Janub Sinai: Wadi Sayal, Gebel El Galala El Bahariya, Near Ain Sayal (USNM 316997); Saint Catherine's Monestary (USNM 317004, 317005, 317006, 317007); Wadi El Sheikh, 3 mi W of Saint Catherine's Monastery (USNM 316998, 316999, 317000, 317001, 317003); Wadi El Arbaeen, Saint Catherine's Monastery Area (USNM 317002). Jordan: 4 Km NW Azraq-Shishan (USNM 429477).

***Arvicanthis niloticus*** (20).—Egypt: Al Fayyum: Faiyum, 3 Mi N Of, Royal Shooting Club (USNM 326069). Al Iskandariyah: Alexandria (USNM 282514). Al Jizah: Abu Rawash, (USNM 300256, 300257, 300258, 326063); Abu Musallam (USNM 326055); Imbaba (USNM 326059, 326060); Sakkara (USNM 300259, 300260, 326056, 326057, 326061, 326062). Matruh, Wadi Natroun (USNM 326068). Minufiya: Ashmun, El Ghunamiya (USNM 326066). Qina, Isna, Wadi Nassim (USNM 326064, 326065). Qualubiya: Qalyub, Qalama (USNM 326067).

***Mus musculus*** (11).—Egypt: Al Jizah, Imbabah District, 2 km east of Abu Rawash (USNM 554205, 554206, 554207, 554208, 554209, 554210, 554211, 554212, 554213, 554214, 554215).
